# Cosmetics for neonates and infants: haptens in products’ composition

**DOI:** 10.1186/s13601-019-0257-8

**Published:** 2019-03-08

**Authors:** Karolina Dumycz, Katarzyna Kunkiel, Wojciech Feleszko

**Affiliations:** Department of Pediatric Pneumology and Allergy, The Medical University of Warsaw, Żwirki i Wigury 63A, 02-091 Warsaw, Poland

**Keywords:** Allergic contact dermatitis, Contact sensitization, Cosmetics, Haptens, Infants, Neonates

## Abstract

Cosmetics and skin care products for neonates and infants are considered as ‘‘hypoallergenic’’, “tested” or ‘‘safe’’. Nevertheless, the prevalence of haptens in these products is a matter of concern, since allergic contact dermatitis in children is gaining an importance. We aimed to assess the prevalence of haptens in cosmetics designed for children younger than 1 year. To identify haptens, the components of the cosmetics listed on packaging were compared with substances from European baseline series, Cosmetics series and Fragrance series. Survey comprised 212 cosmetics among which 186 (87.7%) contained at least one hapten from reference lists. Altogether there were 41 different haptens found in cosmetics. Number of sensitizers per product ranged between 1–12 and, each product contained 2.51 haptens on average. The most abundant sensitizers were cocamidopropyl betaine, tocopherol, propylene glycol, fragrances, lanolin. Majority of products for children were labeled as hypoallergenic/dermatologically tested/safe for children etc. from which 85% contained haptens. This survey highlights the extent of presence of haptens in cosmetics for children under the first year of age.

## To the editor

The skin of neonates and infants, which presents almost excellent appearance at birth, is considered as physiologically fragile with diminished ability to respond to adverse environmental factors [1]. Development of the skin structure and function continues at least until the end of the first year. The function of the skin as a barrier is incomplete at infancy. Studies showed that there was an increased absorption of external substances in infants, mostly due to a thinner epidermis and a higher ratio of skin’s surface area to body weight [1].

Until recently, allergic contact dermatitis (ACD) was considered rare in children, however contact sensitization to haptens has increased over the last decades in children and its prevalence varies between 15 and 71% in children with suspected ACD, depending on the study [2–4]. Although it is accepted that the incidences of ACD increase with increasing age, the disease may be contacted even in early infancy, including sensitization to cosmetic ingredients. Some studies indicate that ACD is significantly prevalent in children between 0 and 4 years of age [2, 3].

The possible risk factors of ACD are skin barrier defects and repetitive or intense exposure to haptens. Delicate skin of neonates and infants require everyday care to maintain proper hydration and purity. Hence, children are exposed to a range of different cosmetics, such as creams, wet wipes, bath products which may contain haptens. In the light of increasing prevalence of ACD in the youngest children awareness of the extent of haptens in cosmetics should rise, especially in health professionals who should properly counsel patients with suspected or diagnosed ACD. Therefore, we aimed to assess to what extent cosmetics for the youngest children contain haptens relevant for cosmetic products.

Between December 2016 and January 2017, two researchers visited 6 different cosmetic stores and supermarkets in Poland and systematically reviewed the shelves and photographed all types of cosmetics for children up to 12 months of age. Our inclusion criteria contained moisturizing agents, bath products, wet wipes, creams and ointments for diaper area, soaps, shampoos and oils. Then, a list of all products was prepared, their ingredients (based on the packaging) using International Nomenclature of Cosmetic Ingredients (INCI) were recorded, then phrases to prove safety or hypoallergenic properties of products were registered. In order to identify the haptens, the components of the analyzed cosmetics were compared to the European baseline series (EBS), Cosmetic series and Fragrance series (Chemotechnique Diagnostics, https://www.chemotechnique.se accessed 13 December 2018) containing 126 haptens in total. Additionally, advertising slogans were compared to the presence of haptens in cosmetics.

Inclusion criteria of our study were met by 212 cosmetics for the youngest children (0-12 months of age). Among the total analyzed products 186 (87.7%) contained at least one hapten, whereas remaining 26 (12.7%) were hapten-free. Further analysis revealed that the most abundant haptens noted were cocamidoporpyl betaine, tocopherol and its esters, phenoxyethanol, fragrances, propylene glycol, ethylhexylglycerin and lanolin alcohols (Table 1). The presence of haptens was assessed in specific forms of cosmetics. We divided cosmetics into three major groups: leave-on, rinse-off and wet wipes. Haptens were present in 86.8%, 89.8% and 84.8% of those groups respectively. Detailed analysis of specific types of products showed highest abundance of haptens in emulsions (100% containing haptens) whereas the lowest number was found in baby oils (66.7%).

**Table 1:** Haptens in cosmetics for infants with numbers and percent of particular hapten

Haptens	Cosmetics containing hapten (out of 212)		Leave-on cosmetics with hapten (out of 91)		Rinse-off cosmetics with hapten (out of 88)		Wet-wipes with hapten (out of 33)	
	No.	%	No.	%	No.	%	No.	%
Cocamidopropyl betaine	65	30.7	0	0	56	63.6	9	27.3
Tocopherol	60	28.3	38	41.8	19	21.6	3	9.1
Phenoxyethanol	55	25.9	23	25.3	15	17.0	17	51.5
Tocopheryl acetate	41	19.3	33	36.3	6	6.8	2	6.1
Propylene glycol	35	16.5	16	17.6	10	11.4	9	27.3
Ethylhexylglycerin	29	13.7	15	16.5	1	1.1	13	39.4
Benzyl alcohol	24	11.3	15	16.5	6	6.8	3	9.1
Limonene	20	9.4	13	14.3	7	8.0	0	0
Lanolin alcohol	19	9.0	15	16.5	3	3.4	1	3.0
Cetyl alcohol	19	9.0	19	20.9	0	0	0	0
Linalool	18	8.5	14	15.4	4	4.5	0	0
Decyl glucoside	17	8.0	0	0	17	19.3	0	0
Paraben mix	12	5.7	7	7.7	4	4.5	1	3.0
Geraniol	12	5.7	11	12.1	1	1.1	0	0
Coumarin	12	5.7	10	11.0	2	2.3	0	0
Methylisothiazolinone	10	4.7	7	7.7	0	0	3	9.1
DMDM hydantoin	10	4.7	1	1.1	9	10.2	0	0
Citronellol	10	4.7	8	8.8	2	2.3	0	0
Sorbitan sesquioleate	6	2.8	6	6.6	0	0	0	0
Citral	6	2.8	5	5.5	1	1.1	0	0
Stearyl alcohol	6	2.8	6	6.6	0	0	0	0
MI/MCI	5	2.4	0	0	5	5.7	0	0
2-Bromo-2-nitropropane-1,3-diol	4	1.9	3	3.3	1	1.1	0	0
Isopropyl myristate	3	1.4	3	3.3	0	0	0	0
Polysorbate 80	3	1.4	2	2.2	0	0	1	3.0
Sorbitan oleate	3	1.4	3	3.3	0	0	0	0
BHA	3	1.4	2	2.2	1	1.1	0	0
Cinnamal	3	1.4	3	3.3	0	0	0	0
Hydroxycitronellal	3	1.4	2	2.2	1	1.1	0	0
Benzyl salicylate	3	1.4	3	3.3	0	0	0	0
HICC	3	1.4	2	2.2	1	1.1	0	0
Chlorhexidine digluconate	2	0.9	2	2.2	0	0	0	0
Propyl gallate	2	0.9	1	1.1	1	1.1	0	0
Cinnamyl alcohol	2	0.9	2	2.2	0	0	0	0
Farnesol	2	0.9	2	2.2	0	0	0	0
Benzyl benzoate	2	0.9	2	2.2	0	0	0	0
Triethanolamine	1	0.5	0	0	1	1.1	0	0
BHT	1	0.5	1	1.1	0	0	0	0
Amyl cinnamal	1	0.5	1	1.1	0	0	0	0
Benzyl cinnamate	1	0.5	1	1.1	0	0	0	0
Butylphenyl methylpropional	1	0.5	0	0	1	1.1	0	0

BHA, 2-tert-butyl-4-methoxyphenol; BHT, 2,6-di-tert-butyl-4-cresol; HICC, hydroxyisohexyl 3-cyclohexene carboxaldehyde; MCI, methylchloroisothiazolinone; MI, methylisothiazolinone

Furthermore, we found that manufacturers used six different marketing terms to prove safety of cosmetics or its “hypoallergenic” compositions (Table 2). Advertising slogan most frequently used was “hypoallergenic”. This term was present in 42% (n = 88) of products identified in our study. Surprisingly, majority of “hypoallergenic” cosmetics contained haptens (89.7%).

**Table 2:** Market terms used by manufacturers to prove safety of cosmetics for newborns and infants

Advertising term	Number of products with advertising term	Percent of products with haptens (n)
Hypoallergenic	88	89.9 (79)
Dermatologically tested	79	88.6 (70)
Recommended by Polish Society of Allergy	20	70 (14)
Positive opinion of National Institute of Mother and Child	8	100 (8)
Dermatologically and allergically tested	8	100 (8)
Safe for children	2	100 (2)
None	7	71.4 (5)

**Fig. 1:**
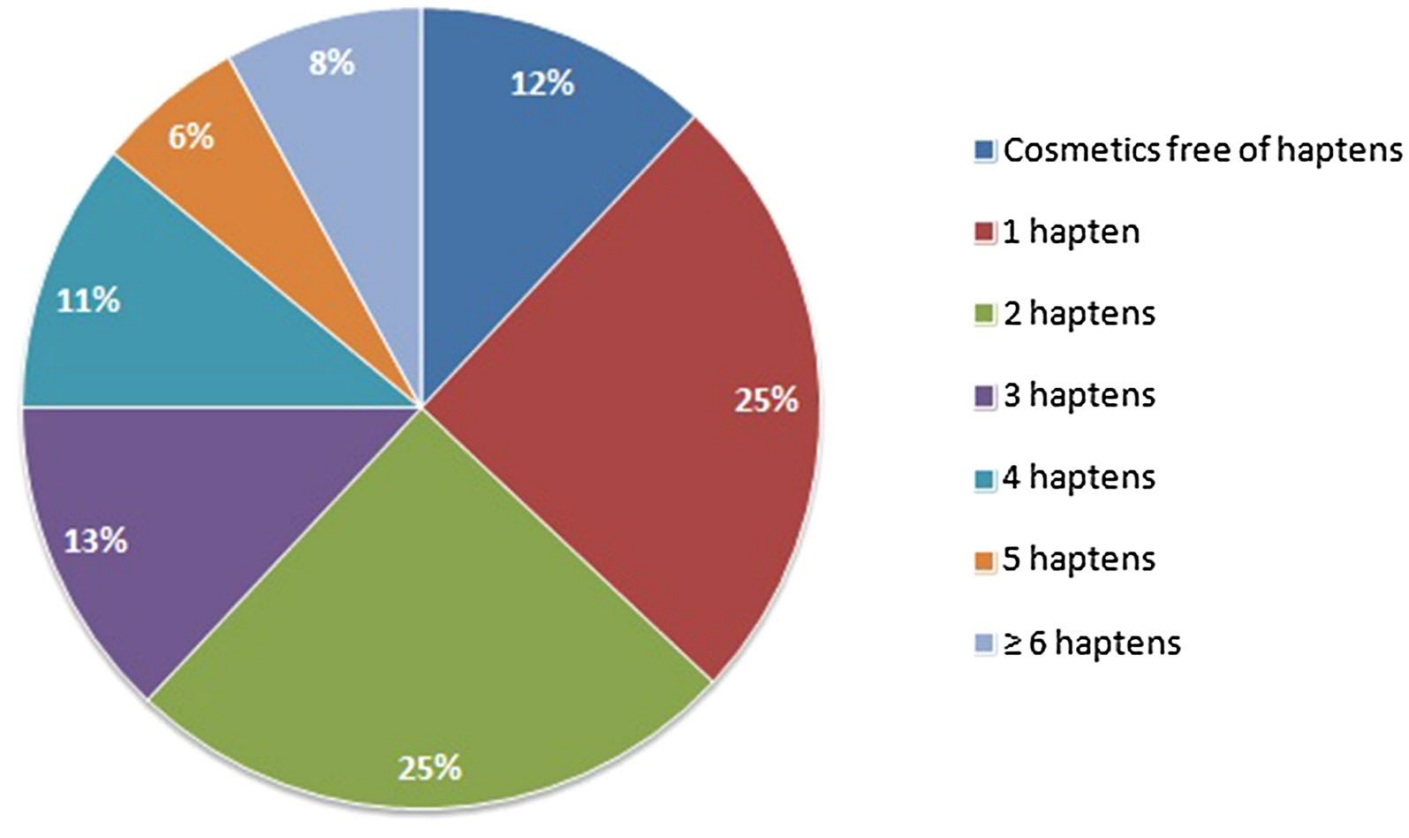
Cosmetics containing different number of haptens

This study reveals high occurrence of potential sensitizers in cosmetics for the youngest children. General percentage of cosmetics containing haptens did not differ significantly between three major groups of cosmetics which were leave-on, rinse-off products and wet-wipes. Haptens were found in 86.8%, 89.8%, 84.8% of products respectively. However, the differences are visible according to particular haptens (Table 1). Worth noting is fact that approximately 37% of all cosmetics contain 3 or more haptens in their composition (Fig. 1).

Similar observations, regarding the high occurrence of haptens in various topical products, were revealed by previous studies [5, 6]. For example, Osinka and colleagues [5] revealed that approximately 60% of cosmetics designed for atopic dermatitis contain substances from EBS. Moreover, Hamann and coworkers [6] reported that 89% of the so-called “hypoallergenic” skin-care products in US contain haptens.

ACD is not rare in the pediatric population. The most abundant haptens in pediatric ACD are nickel sulfate along with cosmetic ingredients such as fragrances and preservatives [2–4]. Therefore it is hypothesized that increase in ACD is mainly be due to the increased exposure to haptens in cosmetic products excessively used in children, toys and clothing. Although the incidence of ACD increases with increasing age, positive results of patch tests may be seen even in infancy, since immature epidermis facilitates early sensitization during the first year of growth leading to greater penetration of haptens [1, 2]. EAACI Task Force on Allergic Contact Dermatitis in Children has also stated that ACD in children appears to be rising. Sensitization to specific substances depends on age and environment. According to authors, most relevant haptens in suspected ACD in children which should be patch tested are nickel sulfate, thiuram mix, colophonium, mercaptobenzothiazole, fragrance mix I and II, mercapto mix, MI/MCI and sesquiterpene lactone mix [7].

In our study the most commonly identified sensitizer was cocamidopropyl betaine found in 30.7% of cosmetics, being present only in rinse-off cosmetics. It is a surfactant which is frequently used in shampoos, soaps and bathing products. Many studies have shown that this chemical is one of the most common causes of ACD in children. Belloni et al. [8] reported that it is responsible for 6.4% of positive patch test reactions in children younger than 3 years. Furthermore, study of Lubbes et al. [3] reveals 15.9% positive reactions to cocamidopropyl betaine.

Fragrance category comprises vast variety of different chemicals. In our analysis we revealed presence of 17 different fragrances in 44 cosmetics in total. Compounds of fragrance mixes I and II are the most frequent sensitizers in pediatric population and are responsible for from 2.5 to 9.9% of positive patch test reactions in the youngest children [4, 9]. Lubbes et al. report that fragrance mixes are among the 5 most common haptens in infants and toddlers (0–4 years). Most frequently found fragrances were limonene and linalool, which are considered as mild sensitizers with a growing rate of ACD in general population [10]. Other commonly found fragrance haptens are geraniol (compound of fragrance mix I) present in 12 of products, coumarin and citronellol (compounds of fragrance mix II), present in 12 and 10 of products respectively. Apart from identified fragrance sensitizers, in 130 of cosmetics only phrase “parfum” was found.

Several cosmetics contained sensitizing preservatives such as MI (methylisothiazolinone) or MI/MCI (methylisothiazolinone/methylchloroisothiazolinone) (10 and 5 products, respectively). These are well-known haptens that have caused numerous ACD cases, that are documented even in the youngest children. Positive patch test reactions to MI/MCI range between 3.3 and 4.4% depending on the study [4, 9, 11]. The sensitization rate was so high that the use of MI/MCI was banned in “leave-on products” in the European Union (European Commission. Consumers: Commission improves safety of cosmetics. http://europa.eu/rapid/press-release_IP-14-1051_en.htm accessed 10 December 2017). Also, MI itself was such a strong sensitizer that its use in the leave-on cosmetics and wet wipes was banned with a deadline on 12 February 2017 in the European Union (European Parliament. Annex V of the Regulation (EC) No 1223/2009 of the European Parliament and of the Council on cosmetic products. https://eur-lex.europa.eu/legal-content/EN/TXT/?uri=CELEX%3A32016R1198 accessed 10 December 2017) and restricted in rinse-off products from 100 to 15 ppm (parts per million) (European Commission. Annex V to Regulation (EC) No. 1223/2009 of the European Parliament and of the Council on cosmetic products. https://eur-lex.europa.eu/legal-content/EN/TXT/?qid=1551781055624&uri=CELEX:32017R1224 accessed 12 December 2017).

Lanolin, which can be found in 9% of cosmetics, causes ACD especially in the youngest children with sensitization rate between 13 and 1.5% [3, 4]. Despite of potential sensitization properties lanolin still will be widely used in cosmetics because of its functionality as an emollient.

Presence of propylene glycol in 16.5% of cosmetic products was reported. Although it is rare cause of ACD in children it is frequently responsible for irritant contact dermatitis. Hence, this should not be used in children under the age of two.

In this research high frequency of tocopherol (28.3%), phenoxyethanol (25.9%), tocopheryl acetate (19.3%), ethylhexylglycerin (13.7%), benzyl alcohol (11.3%) and cetyl alcohol (9%) in cosmetic composition was noted. Despite proven sensitizing properties these haptens seem to have low significance in ACD in young children.

An important limitation of this research is that the cosmetics’ base has been limited only to those available in the six largest cosmetics stores, where these products are easily available to consumers. Less popular brands of cosmetics may only be available through the online store, so the list of products may not be representative of the entire market of cosmetics for neonates and infants. Another limitation is that our analysis was carried out only in one country. However, due to common legislation within the European Union, we assume that the observed phenomenon can be generalized and that a similar situation could be found in other European countries. Indeed, a recent analysis from Denmark revealed that fragrance haptens were found in total 49 out of 230 products for children [10], whereas in our analysis 44 out of 212 cosmetics comprised at least one fragrance hapten. Furthermore, we neither assessed the actual concentration of haptens in cosmetics nor the actual exposure of children to haptens by cosmetics. In addition this survey is limited to labeling of haptens in products for infants and does not assess prevalence of ACD to those substances in children.

Current analysis reveals that most of skincare products for neonates and infants contain haptens present in EBS, Cosmetic series or Fragrance series. This study allows to identify products that should be avoided in children with diagnosed ACD. However, whether avoidance of haptens by infants with immature skin barrier is a proper strategy for primary prevention of ACD is unknown. Moreover, there is still no directive in the European Union that defines and distinguishes the terms “hypoallergenic” and “free from”, therefore an action should be taken to regulate the use of such terms.

## Abbreviations

ACD: allergic contact dermatitis
EBS: European baseline series
INCI: International Nomenclature of Cosmetic Ingredients
MCI: methylchloroisothiazolinone
MI: methylisothiazolinone
